# Familial Occurrence of Isolated Late-onset Nasolacrimal Duct Obstruction in Two Unrelated Families

**DOI:** 10.5041/RMMJ.10519

**Published:** 2024-01-19

**Authors:** Anat Bahat Dinur, Ortal Buchbut, Libe Gradstein, Baker Elsana, Ofek Freund, Ohad S. Birk, Erez Tsumi

**Affiliations:** 1 Department of Otolaryngology & Head and Neck Surgery, Soroka University Medical Center and Clalit Health Services, The Faculty of Health Sciences, Ben Gurion University of the Negev, Beer-Sheva, Israel; 2 Department of Ophthalmology, Soroka University Medical Center and Clalit Health Services, The Faculty of Health Sciences, Ben Gurion University of the Negev, Beer-Sheva, Israel; 3 The Morris Kahn Laboratory of Human Genetics, National Institute for Biotechnology in the Negev, Beer-Sheva, Israel; 4 Genetics Institute, Soroka University Medical Center, Ben-Gurion University of the Negev, Beer-Sheva, Israel

**Keywords:** Endo-DCR, epiphora, nasolacrimal duct obstruction, oculoplastic

## Abstract

Late-onset nasolacrimal duct obstruction (NLDO) as a result of inflammatory processes causing dacryo-stenosis is a common entity affecting mostly women. While a few mechanisms have been suggested as contributors to the expression of NLDO, the trigger for the inflammation remains mostly unknown. Familial predilection for this condition has not been previously reported. We present two families with multiple individuals affected with congenital or late-onset NLDO, describe the signs and symptoms of the affected individuals, and explore their medical history for any contributing factors. Family A, spanning four generations, included 7 female patients affected by late-onset NLDO. Family B, spanning two generations, included 8 individuals affected by either congenital or late-onset NLDO. This case series suggests a familial predisposition to NLDO, apparently with an autosomal dominant inheritance pattern. Further studies are needed to elucidate the molecular basis of this genetic predisposition.

## INTRODUCTION

Nasolacrimal duct obstruction (NLDO) is associated with epiphora and recurrent dacryocystitis and usu-ally requires surgical intervention. The congenital form (CNLDO) is caused by incomplete perinatal perforation of the membrane present at the nasolac-rimal duct opening. Late-onset disease is thought to be associated with obstruction along the nasolacrimal duct and is subdivided into primary and secondary acquired nasolacrimal duct obstruction (PANDO and SANDO, respectively).^1^ Primary acquired NLDO is caused by inflammation and fibrosis of the naso-lacrimal system without any known precipitating cause, although anatomical and hormonal predis-posing factors have been suggested.^2^–^6^ The smaller bony diameter of the lacrimal duct or reduced ex-pression of estrogen receptors in lacrimal mucosa is more pronounced in women than in men, contrib-uting to the higher incidence of PANDO in females. Of all the non-traumatic forms of dacryostenosis, PANDO accounts for most of the cases observed in adults. The secondary form is diagnosed when the patient’s history indicates likely causes such as trauma, sinus disease, sinus surgery, or systemic dis-eases.^1^ The exact incidence of late-onset NLDO is not known, although it is considered quite common.^7^

Familial occurrence of CNLDO has been reported previously. In most of these cases, CNLDO occurred as part of a systemic syndrome or was associated with various developmental anomalies. Little is known regarding the inheritance pattern of isolated CNLDO. Several studies have suggested familial occurrence of isolated CNLDO with an autosomal dominant pedigree pattern, but the genetic basis has not been identified.^8^ Only one germline mutation associated with CNLDO has been reported to date. Foster et al. identified a homozygous IGSF3 muta-tion in a consanguineous family with CNLDO.^9^

To the best of our knowledge, there have been no reports of familial occurrence of late-onset NLDO. Here we report two large unrelated Israeli Jewish families of Moroccan and Ashkenazi ancestry with several members affected by isolated late-onset NLDO. In one of the families, both CNLDO and late-onset NLDO were found. We describe the clinical features of the disease in these two families.

## THE CLINICAL CASE SERIES

Several patients from two unrelated families of Israeli Jewish ancestry presented to the oculoplastic clinic in the ophthalmology department at Soroka University Medical Center (SUMC), Beer-Sheva, Israel, with similar complaints of epiphora. Their pedigrees were obtained, and they underwent com-plete ophthalmic examination. General medical his-tory was reviewed, and systemic symptoms and signs were recorded. Clinical data, including demograph-ics, clinical presentation, systemic and ocular dis-orders, and treatment were collected for all affected individuals. The diagnosis of PANDO and CNLDO was made by history and physical examination, as well as by Jones tests, when needed. Study of this case series was approved by SUMC Institutional Review Board and Ethics Committee and adhered to the tenets of the Declaration of Helsinki.

### Family A

**Figure 1:**
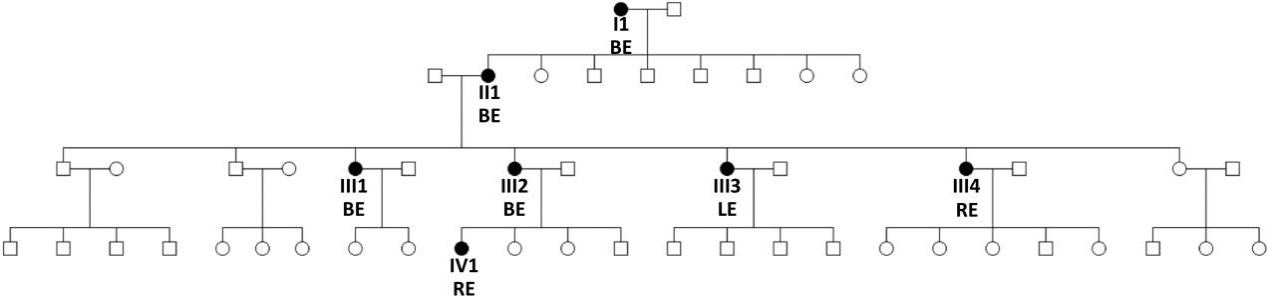
Family A Pedigree Squares represent males; circles represent females; black filled objects indicate individuals with late-onset nasolacrimal duct obstruction. Subject numbers correspond with those used in Table 1. BE, both eyes; I, first generation; II, second generation; III, third generation; IV, fourth generation; LE, left eye; RE, right eye.

Family A included 7 female patients with late-onset NLDO spanning four generations (Figure 1). This non-consanguineous family was of Moroccan Jewish ancestry. All females diagnosed with late-onset NLDO were daughters of mothers also affected by the condition. The age of onset ranged from 18 to 40 years, with the presenting sign in all cases being epiphora (Table 1). Most affected individuals had no history of chronic ocular or periocular inflamma-tion, sinus inflammatory disease, or systemic abnor-malities. Most affected individuals had normal find-ings on ophthalmic examination and no sinonasal pathology except NLDO. Blepharitis and cataract were diagnosed in two different subjects; however, these conditions are not known to be associated with NLDO. None of the patients had ptosis, facial dys-morphism, or abnormalities of eye movements. Two subjects had a history of sinusitis, but no correlation was found between sinusitis onset and epiphora exacerbations. Surgical intervention was necessary for NLDO treatment in all but one patient. A com-puted tomography scan of the orbits in one patient revealed normal results, showing no pathological or contributing factors to the obstruction.

**Table 1:** Family A: Demographics and Medical History of Affected Individuals.

Parameter	Subject Number						
	I1	II1	III1	III2	III3	III4	IV1
Sex	F	F	F	F	F	F	F
Age at onset (years)	40	40	22	18	37	32	25
Eye involved	BE	BE	BE	BE	LE	RE	RE
Epiphora	Y	Y	Y	Y	Y	Y	Y
Dacryocystitis	N	N	N	Y	Y	Y	N
Other ocular conditions	N	N	N	Blepharitis	N	Cataract	N
Topical eye treatment	N	Tobramycin	N	N	N	N	N
Smoking	N	N	N	N	N	N	N
Other sinonasal disorders	N	Chronic rhinitis Acute sinusitis	N	N	N	N	Chronic sinusitis
Other systemic diseases	DM	N	N	N	N	N	N
Treatment	Conservative	RE: EXT-DCR LE: ENDO-DCR	LE: EXT-DCR RE: EXT-DCR RE: ENDO-DCR RE: ENDO-DCR*	RE: ENDO-DCR LE: EXT-DCR	LE: ENDO-DCR	RE: ENDO-DCR	RE: ENDO-DCR

BE, both eyes; DES, dry eye syndrome; DM, diabetes mellitus; ENDO-DCR, endoscopic dacryocystorhinostomy; EXT-DCR, external dacryocystorhinostomy; F, female; I, first generation; II, second generation; III, third generation; IV, fourth generation; LE, left eye; M, male; N, no; RE, right eye; Y, yes.

* Repeat procedure at different times after failure and return of epiphora.

### Family B

**Figure 2:**
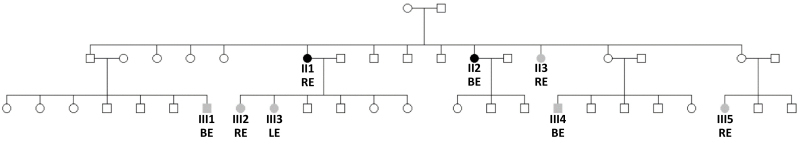
Family B Pedigree Squares represent males; circles represent females; black filled objects indicate individuals with late-onset nasolacrimal duct obstruction; gray filled objects indicate individuals with congenital nasolacrimal duct obstruction. Subject numbers correspond with those used in Table 2. BE, both eyes; II, second generation; III, third generation; LE, left eye; RE, right eye.

Family B, of non-consanguineous Ashkenazi ances-try, included 8 affected individuals: 2 female pa-tients in generation II were affected by late-onset NLDO, 1 patient in generation II was affected by CNLDO, and 2 male and 3 female patients in gener-ation III were affected by CNLDO which spontane-ously resolved with age (Figure 2). No predisposing factors to NLDO were noted (Table 2). Among the CNLDO cases, two were confirmed and recorded by a physician, and the other four were reported by parents.

**Table 2:** Family B: Demographics and Medical History of Affected Individuals.

Parameter	Subject Number							
	II1	II2	II3	III1*	III2*	III3*	III4*	III5
Sex	F	F	F	M	F	F	M	F
Age at onset	36 yr	16 yr	5 yr	6 mo	6 mo	1 yr	6 mo	4 yr
Eye involved	RE	BE	RE	BE	RE	LE	BE	RE
Epiphora	Y	Y	Y	Y	Y	Y	Y	Y
Dacryocystitis	N	Y	N	N	N	N	N	N
Other ocular conditions	N	N	N	N	N	N	N	N
Topical eye treatment	Ofloxacin Dexamethasone	Ofloxacin Dexamethasone	N	N	N	N	N	N
Smoking	N	N	N	N	N	N	N	N
Other sinonasal disorders	N	N	N	N	N	N	N	N
Other systemic diseases	N	N	N	N	N	N	N	N
Treatment	LE:ENDO-DCR	LE:ENDO-DCR	Conservative	N	N	N	N	N
Comments	N	N	Down syndrome, CNLDO	Epiphora, CNLDO*	Epiphora, CNLDO*	Epiphora, CNLDO*	Epiphora, CNLDO*	Epiphora, CNLDO

BE, both eyes; CNLDO, congenital nasolacrimal duct obstruction; DES, dry eye syndrome; DM, diabetes mellitus; ENDO-DCR, endoscopic dacryocystorhinostomy; F, female; II, second generation; III, third generation; LE, left eye; M, male; mo, month; N, no; RE, right eye; Y, yes; yr, year.

* Reported by parents, spontaneous resolution noted with age.

## DISCUSSION

We report two unrelated extended families with multiple members affected by late-onset NLDO. The absence of associated systemic conditions or local risk factors for NLDO implies a genetic predispose-tion. While familial cases of CNLDO have been traced, to the best of our knowledge, this is the first report of familial occurrence of late onset NLDO.

The incidence of PANDO is greater in women than in men.^1^ This higher occurrence in females is thought to be the outcome of significantly smaller dimensions of the lower nasolacrimal fossa and mid-dle nasolacrimal duct. Groessl and colleagues noted that changes in the anteroposterior dimensions of the bony nasolacrimal canal coincide with osteo-porotic changes throughout the body.^2^ Others have suggested menstrual and hormonal fluctuations and augmented immune status as factors that may con-tribute to the disease process.^3^–^6^ These may explain the high prevalence of PANDO in middle-aged and elderly women, when the hormonal changes leading to generalized de-epithelialization in the body may cause the same process within the lacrimal sac and duct.^1^ It is possible that an anomalous nasolaryngeal duct structure is inherited in an autosomal domi-nant fashion in both sexes, but the already narrow lacrimal fossa in women predisposes them to ob-struction by sloughed-off debris, thereby manifest-ing as overt NLDO only in affected females. The fact that only female family members in Family A were affected with NLDO could also imply a non-Mendelian mode of inheritance. In Family B, how-ever, two male children were affected by CNLDO. Moreover, three CNLDO patients had mothers with no history of NLDO. Therefore, if late-onset NLDO and CNLDO are considered as the same entity, the most likely mode of heredity is autosomal dominant with incomplete penetrance and variable expression.

The occurrence of both late-onset NLDO and CNLDO in Family B could imply that late-onset NLDO might be a form of occult CNLDO that be-comes clinically apparent after puberty. Affected individuals in Family B developed a more severe, congenital disease in the younger generation as opposed to the milder, adult-onset disease in older individuals, which may indicate the mechanism of genetic anticipation. It should be mentioned that while a few studies suggested familial occurrence of congenital dacryocystocele and CNLDO, a clear in-heritance pattern has not been reported, except for the association with a homozygous IGSF3 variation in one consanguineous family with CNLDO.^9^

In summary, to the best of our knowledge, this is the first report of familial occurrence in two un-related non-consanguineous families of late-onset NLDO, with likely autosomal dominant heredity with incomplete penetrance and variable expression. Further studies are needed to elucidate the molecu-lar basis of this genetic predisposition.

## Abbreviations

CNLDO: congenital nasolacrimal duct obstruction
NLDO: nasolacrimal duct obstruction
PANDO: primary acquired nasolacrimal duct obstruction
SANDO: secondary acquired nasolacrimal duct obstruction.
